# Current Notes and Abstracts

**Published:** 1880-01

**Authors:** 


### CURRENT NOTES AND ABSTRACTS.

Remarkable Epizootic in Venezuela.—A strange disease broke out among the immense herds on the Llanos of Venezuela in 1843, to which about seven million horses, mules, and asses fell victims. The chief feature was the appearance of paraplegia, the general condition being very good; but, as the paralysis showed no tendency to recovery, the animals were killed, as useless. The most interesting feature of the disease was, that it also attacked the indigenous monkeys, capybaras, et cetera, while it did not affect horned cattle in the slightest. The appearance of the epizootic was inaugurated, and probably caused, by a terrible mortality of the water animals.

Large numbers of dead crocodiles and fish floated down the rivers, and putrid fevers proved fatal to human inhabitants. The disease, in a milder form, still exists among horses.—*Sachs: Reise nach Venezuela.*

---

Epidemic Abortion of Cows.—Frank considers the cause of this puzzling affection to lie in some infectious principle contained in the vaginal mucus of already aborted cows, which is known, when communicated to the vagina of a healthy cow with calf, to produce abortion in her. He recommends, as a prophylactic, the isolation of the cows which have aborted, and the removal of the healthy ones, when possible, to another locality.—*Deutsch: Arch. f. Thiermed. u. Vergl. Path. III.*

---

Parasitic Bronchitis in Dogs.—Oster found that an affection of chiefly young dogs, beginning with loss ef appetite, ataxic gait, or even paraplegiform weakness, rise of temperature and little or no cough, and which terminated in death either by gradual exhaustion, or with vomiting and convulsions, was due to the presence of a nematode parasite, which he terms *Strongylus canis bronchialis* The body of the worm is thread-shaped, and the female, which is a quarter of an inch long, has living young. The worms lie either on the surface of the bronchial mucous membrane, or, as more frequently is the case, are encapsuled in nodular thickenings of the latter. No explanation for their mode of migration from one dog to the other was discovered.—*The Veterinarian*, June, '77.

---

Monstrosities Due to the Paternal Influence.—Rost describes a series of ten monstrosities in calves, which all were born on the same farm by seven different cows. One of these was primiparous, the others had well-formed calves previous to giving birth to monstrosities. The mal-formed calves had supernumerary ears and a double hyoid arch, and most of them died in the course of five days with clonic spasms. It was found that the monstrosities had only happened after the introduction of a certain apparently well-formed bull. The latter was excluded from the farm, and the seven cows mentioned produced normal calves.—*Rost: Bericht über das Veterinarwesen im Königreich Sachsen f. d. J. 1876.*

---

Detachment of the Retina in Horses.—Berlin states that veterinarians have been accustomed to consider as glaucoma every case where the eye presents a greenish reflex, while he has found, on examining such eyes, that the actual lesion was retinal detachment. True glaucoma has not thus far been observed in the horse.

---

Growth and Development of the Tapeworm.—Perroncito administered a Coenurus cerebralis from a sheep's brain to a dog. After five months the dog began to void taenia proglottides, and, on being killed a month later, fifty-nine specimens of taenia coemurus were found, the larger ones measuring over a yard in length, and possessing up to twenty-five mature links. A pupil of Perroncito (who must have possessed an unenviable heroism) swallowed a cysticercus from the calf. After fifty-four days he began to pass ripe proglottides, and on the seventy-six day, after a vermifuge, he discharged a taenia measuring over four feet in length (4,774 M.). From this Perroncito calculates the daily growth of the taenia at two inches, or thirteen links.—*Zeitschrift für Veterinarwissenschaften. Bern, V.*

MONSTROSITIES OF OVA IN CHICKENS.—Landois, in the course of an exhaustive series of researches, has collected about one hundred and fifty varieties of deformed eggs. The largest eggs measured from 77 to 83 millimeters in length and 48 to 52 in width. The smallest ripe egg measured 14 millimeters by 10, (half an inch by 4-10 inch.) He found, quite in contradiction with the English embryologist Balfour, that the blunt egg-end is the *first* to leave the cloaca. He found *eggs enclosing other eggs*, and what is strangest of all, foreign bodies, such as beetles' legs, in the albumen. Very small eggs contained albumen only. Some chickens laid nothing but double-yolked eggs all the year round. Screw-shaped eggs proved that the movement of the eggs through the oviduct occurs by a spiral movement. – *Zoölogischer Garten, Nr. 1.*

---

LEUCOCYTHAEMIA IN DOMESTIC ANIMALS.—A six-months pig was found to have all the lymphatics, especially of the mesentery, enlarged; besides this, leukaemic lymphomas scattered over the peritonal surfaces, and leukaemic infiltration of the bronchi. The proportion of white to red corpuscles was as 1 : 2-4. Duration of disease, four weeks. While the form of the Leukaemia was essentially lymphatic here, it was splenic in a second case—a pig of the same age. The spleen in the latter was enlarged to a length of 17 and a width of 2½ inches, showing numerous white infiltrations. Death occurred by cerebral hemorrhage in a third case, a dog of three years, in whom the liver, spleen, bronchial glands and the medulla of the bones exhibited characteristic lesions. The fourth case, a cow, showed lymphoid infiltration of the uterus and broad ligaments; proportion of leucocytes, 1 : 10-15.—*Siedamgrotzky: Bericht für das Königreich Sachsen f. d. J. 1876.*

---

PREVENTION OF TRICHINOSIS IN SWINE.—It being now well established that trichina in the hog are produced by the habit which this animal has of devouring rats (usually captured at night). It follows that all pig-pens should have solid walls and cement flooring. No pigs should be permitted in the vicinity of knackeries, mills, or slaughter-houses. One of the chief sources for the propagation of trichinosis is the fact that knackers keep pigs to feed them on animal offal. Therefore knackeries should not be permitted to keep pigs.—*Eulenberg: Vierteljahrschrift für gerichtliche Medizin und öffentliches Sanitätswesen, Bd. XXVIII.*

---

HAEMATURIA IN THE ROEBUCK.—Anacker found that an epizootic of haematuria among roebucks was due to their feeding on acid plants, which produced renal irritation.—*Thierarzt, 1.*

---

STOMATITIS PUSTULOSA CONTAGIOSA OF THE HORSE.—This disease has its chief seat in the mouth, as well as on the skin around the mouth and nostrils. First small inflammatory nodules are observed, which become first pustular, and are then converted into ulcers. *At this latter stage it may easily be confounded with glanders.* The disease may be transferred to cattle, sheep, pigs, and even to man. The contagion-bearer is the saliva. The tendency is to spontaneous cure.—*Eggeling und Ellenberger: Archiv für Thierheilkunde, Bd. IV.*

RACHITIS IN PIGS.—Utz claims that this disease has become more frequent since the introduction of English breeds into Germany, and thinks overfeeding with potatoes is an exciting cause. Pigs that are allowed to run wild are rarely affected. He recommends bone-meal as a remedy.—*Utz: Thierärztliche Mittheilungen, XII.*

---

CONTAGIOUS PLEURO-PNEUMONIA IN THE WILD BOVINE.—Two Siberian Yaks (*Bos grunnicus*), three American bisons, and one European buffalo became attacked by pleuro-pneumonia in the zoölogical gardens of Brussels. All perished of the disease except the buffalo, who was killed. The autopsy showed the usual changes of contagious pleuro-pneumonia.—*Veröffentlichungen des kais. deutsch. Gesundheits-amtes.*

---

DIAGNOSIS OF GLANDERS.—One of the most certain means to determine the existence of glanders is the removal of one or two of the peri-laryngeal lymphatic glands, which, if the horse is glandered, will exhibit miliary nodules of a dirty-yellow color, and miliary abscesses.—*Bollinger: Wochenschrift für Thierheilkunde, ü. s. w...22. Jahrgang.*

As illustrating the liabilities to error in the recognition of glanders, Anacker reports a case of ulcerative Angioma of the *septum narium*, which during life had exhibited running from the nose, swelling of the submaxillary glands, and other symptoms usually considered to justify a diagnosis of glanders.—*Anacker: Thierarzt, Nr. 1.*

---

PATHOLOGY OF "PIG-TYPHOID."—The skin is injected and reddened, and in circumscribed places gangrenous. The mucous membrane of the small intestines is ecchymotic, and that of the large intestine is the seat of ulcers. These ulcerations, however, are not analogous to those found in the typhoid fever of the human subject, for while in the latter they are seated in the lymphoid glands (Peyer's patches and the solitary follicles), they are in the pig in no relation whatever to these structures. The peripheral lymphatic glands are swollen and discolored by bloody infiltrations, as in anthrax. Besides this, pericarditis, pleuro-pneumonia, and peritonitis are found to be frequent complications. The spleen is swollen in some cases, the liver enlarged, and hyperaemic in all. The kidneys are hyperaemic, and often the seat of small hemorrhages. There are also superficial ulcerations of the oral pharyngeal mucous membrane. On microscopic examination, the ulcerations of the intestinal tract and mouth are found, like those of the skin, to be essentially necrotic. No anthrax-bacilli being found, the disease is to be regarded as having nothing essential in common with anthrax. The disease is infectious, however, and the contagion seems to reside in the skin.—*E. Klein: Experimental and Anatomical Inquiry into the so called Pig-Typhoid. Trans. of the Path. Soc., XXVIII.*

---

PATHOLOGY OF THE NERVE-CENTRES IN HYDROPHOBIA.—Forel examined microscopically more than 450 microscopic sections from animals and one human being that had died of hydrophobia, and failed to find anything pathognomonic, especially finding nothing even remotely corresponding to the highly colored statements of Benedict, who found multiple abscesses (!), pigment clumps, and so on. All the appearances found could be referred to secondary factors, such as the hyperaemia.—*Forel: Deutsches Archiv für Thiermedizin u. Vergl. Path. III.*

EQUINE TYPHUS.—A destructive epidemic broke out in Syria during the spring of 1876, and was conveyed by the horses of the "Baschi-Bazuks" to the troops collected near Suez from Abyssinia and Egypt. Wherever in their march the cavalry stopped, the epidemic gained a foothold, and even reached the horses of the Desert-Beduins. In and around Cairo 5,400 horses and mules died of the affection, and in one of the provincial districts, Cherkieh, 4,700 fell victims. There was not the slightest attempt at quarantine, no isolation of the infected stables, not even the bodies of the dead animals were buried. The Egyptian veterinarians betrayed an ignorance and stupidity which would almost appear incredible, had we not seen in the opponents of the quarantine regulations, directed by Professor Law, during the recent pleuro-pneumonia endemic, the worthy colleagues of these Egyptians even in our own country. Drs. Sonzino and Ball, of Zagazig and Cairo respectively, describe the symptoms as following each other in two stages. The first beginning with loss of appetite, drooping of the head, stupor, tremulous and ataxic movements, and general weakness. The temperature varies between 39° and 40° Centigrade. Conjunctiva swollen and injected, occasionally covered with a purulent exudation. Tongue hot, tumefied, and covered, especially on the apex and sides, with hemorrhagic spots, which may extend to veritable hemorrhages.

This febrile stage is followed by collapse. The skin becomes unnaturally cool after the previous febrile heat. Respiration becomes short, accelerated, and abdominal; pulse small, and almost imperceptible. The animals die in a protracted agony of several hours, during which the respiration becomes more and more difficult and interrupted.

It is a very furibund affection; it may kill in a few hours. The longest duration is from two to three days. Cases of recovery were rare, and recovery was never the result of any special treatment; but the fact that such a case had been originally a mild one, that is more or less abortive. Autopsies showed that there were ecchymoses of all the serous and mucous membranes and enlargements of the various glandular organs.—*Veröffentlichüngen des Kaiserlich Deutschen Reichsgesundheitsamtes. Beilage, Nr. 6, I.*

---

THE RINDERPEST IN GERMANY.—During the first three months of the year 1877, 94 head of cattle died of this affection, and 1238 head of cattle, 337 sheep and 9 goats were slaughtered by governmental order, and the meat of those so slaughtered *utilized.* One of the most instructive lessons derived from this epidemic was, that the markets for cattle to be slaughtered in large cities exert a most potent influence in the propagation of this formidable disease.—*Müller: Berlin. Archiv, p. 481, 3d Vol.*

The same epidemic was the occasion of a thorough and exhaustive investigation by the German Imperial Health-Office. The infection was traced to cattle which had been smuggled over the Prussian frontier, and had thus evaded sanitary inspection. The infected markets being promptly closed, and all the cattle therein slaughtered, it was possible to isolate and stamp out the affection. It was shown that the contagion is not usually carried from place to place by patently diseased, but by apparently healthy, though infected animals. While the infection of far distant localities implied the transportation of the diseased cattle themselves, it was evident that neighboring villages and towns became infected through intermediate contagionbearers, such as human beings. The latter act so exceptionally, however, in many instances, veterinary empirics who treated cattle in different localities and came in daily contact with the disease, failed to transport it to all the localities visited by them. The stamping out process was shown by the history of this speedily suppressed epidemic to be the most efficacious.

Two shiploads of apparently healthy cattle, from Hamburg, carried the disease to England, where 363 cattle became attacked, of whom 35 died and 228 were killed. In addition, 835 healthy cattle in the infected localities were killed, and thus an epizootic, which might otherwise have destroyed millions on millions of property, was suppressed, at a cost to the government of £13,000 sterling.

In Russia, whose defective veterinary and sanitary regulations have been not unjustly accused as the cause of the frequent outbreaks of the Rinderpest in Europe, it has been found that since the stamping out process has been resorted to in certain provinces, that the disease has become much rarer. The loss in cattle was previously one per hundred head; it is now one per thousand.—*Schmulewitsch : Wochenschrift für Thierheilkünde, ü. a. m., 22. Jahrgang, 1878.*

---

ANTHRAX.—The reports of the Prussian veterinarians show that, during the prevalence of the epidemic of 1876—1877, 39 human beings were attacked by anthrax, 13 of whom died. It was a remarkable feature of this epidemic that it attacked wild animals also. Thus, of the 600 deer in the forest of Primkenau, 269 perished of this affection.—*Reports of the* " *K. Gesündheitsamt.*"

---

PATHOLOGICAL COURSE OF ANTHRAX.—Colin finds that the lymphatic glands are the first points for accumulation of the virulent agent, and the glands become affected in the order of their anatomical arrangement. For the whole term of the disease these glands act as depots for repeated infection of the system, and the infectious property resides in them before the existence of bacteria can be proven. The appearance of the bacteria, however, signalizes a more rapid progress of the affection.—*Bulletin de l Academie de Mediçine, No. 30, 1878.*

---

EMPHYSEMA CONTAGIOSUM.—Bollinger, the indefatigable pathologist of the Munich Veterinary School, reports the existence of a remarkable affection in the mountain districts of Bavaria, which, ordinarily designated as "Rauschbrand," "Milzbrand-emphysem," he proposes to term contagious emphysema. It usually attacks cattle in those districts in which "Milzbrand" (anthrax) is frequent of occurrence. In some places, from one to five per cent. of the cattle perish annually of this disease, which is characterized by a highly acute emphysema of the subcutaneous cellular tissue and of the muscles, associated with a sero-hemorrhaginous infiltration, most marked in the thigh. The emphysema extends over the greater part of the animal, and kills in from one to two days. It has never been known to affect man or other animals than the cow. In a series of inoculations performed by Bollinger he found that the disease could readily be transferred to cattle, sheep and goats, the inoculated animals dying in from twenty to thirty hours. The emphysema developed primarily around the spot of inoculation. The gas found in the cellular tissue burned with a blue flame and was found to have about the composition of marsh-gas ($CH_4$). The blood was found full of gas-bubbles—the lymphatics, filled by similar bubbles, giving them the appearance of rows of glass beads. *Bacilli* were found in the blood, but strangely enough decomposition in the cadaver was not unusually rapid. On infecting a calf with the same material by the mouth, it died without either peripheral or intestinal emphysema, although *bacilli* were found in the blood.—*Bollinger: Bayr. Aerztliches Intelligenzblatt, Nr. 26, 1878.*

---

A New Fungoid Disease in Cattle.—Bollinger, the distinguished veterinary teacher of Munich, who has done great service to his profession in rendering clear affections, which, under different and misleading terms, had long been a source of confusion to the student, now appears as the founder of another, hitherto unnamed or misnamed affection, for which, with his usual acumen, he establishes a clear pathogeny. The disease, like many other affections of cattle, has passed under the aliases of Osteosarcoma, Spina ventosa, Knochenwurm, bone cancer, chronic ostitis, and tubercle of the osseous structures. It manifests itself in tumefactions of the premaxillae and the great supramaxilla, destroying the bone and leading to ulceration, formation of abscesses, and fistulous tracts. At the same time the tongue becomes the seat of peculiar tuberculated nodules, and other changes, which give this organ a peculiar wood-like solidity, so that the German farmers term such tongues "Holzzüngen." The lymphatic glands of the submaxillary glands are also frequently enlarged.

Both in the bony new formation, the tongue and the lymphatics, Bollinger found an endophytic fungus; it was equally found in similar growths of the larynx, pharynx, and gastric mucous membrane. The fungus has a mulberry shape; on pressure, it falls into several wedge-shaped fragments, each of which is an individual fungus, so that the whole is a fungoid colony. The fungus is named *Actinomyces bovis.* It is found in the cheesy, disintegrated tissue which fills the spongy framework of the new formations.—*Bollinger: Ueber eine neue Pilzkrankheit beim Rinde. Centralblatt f. d. Med, Wiss., Nr. 27, 1877.*

---

Atrophy of the Pancreas in a Cow.—A cow with chronic, incurable diarrhœa was found to be suffering from atrophy of the pancreas, referable to pancreatic calculi.—*Wochenschrift für Thierheilkunde und Viehzucht, XXI.*

---

Evil Effects of Milk from Diseased Cattle.—In Strassburg it was observed that children who drank *unboiled* milk from cattle afflicted with the foot and mouth disease, slight *malaise*, vomiting and aphthous eruption in the mouth followed.—*Veterinary Reports for Alsace-Lorraine, 1877—1878.*

---

Veterinary Experience with the Camels Used in the British Campaign in Afghanistan.—Mr. Charles Steele, Veterinary Surgeon to the 17th Lancers, in a very extensive paper, presents his experience of the camel during the memorable Afghan campaign. He seems to imply that very little was done by the powers that be to maintain the camel-service at its most effective condition, and if his charges are true, and they seem to be made by one possessing both earnestness of purpose and good practical sense, much red tape must still exist in the management of an important military department. We say important, because from the great and avoidable mortality of camels, the column of Col. McLeod, destined to carry ammunition to the advanced corps, failed in its object. It lost, out of eight hundred camels, with which this column started, over *six hundred* animals. Acute pneumonia, contracted in the high and cold passes of the Khyber and Kurum valleys, was the most frequent cause of death. A remarkable feature of this pneumonia was, that, as shown by post-mortems, the pleura was not coincidently affected, which is an evidently exceptional feature with the camel A chronic pneumonia also occurred, induced by combined exposure and privation, and which Mr. Kettlewell, Veterinary Surgeon to the Saharumpore stud, believes to be scorbutic in nature. Among the avoidable causes of loss were—1st, the employment of too young camels; 2d, defective arrangements for proper feeding; 3d, improper over-driving; 4th, wilful neglect by the Serwans, who, knowing that they would be indemnified by government for such camels that died, wilfully permitted them to die, finding a great load off their minds when rid of the camel; 5th, over-credence to the current popular idea, that the camel can go an unlimited period without water.—*Camels in connection with the South Afghan Expedition, 1878—1879; by Charles Steele, V. S. 16th Lancers, employed on special service. The Veterinarian, Oct., 1879.*

---

Editorial Comment on Above: Accompanying this paper are some interesting anatomical and physiological remarks, some of which, however, the writer seems to generalize too much on. He, for example, considers it an error that the camel can go, without being seriously affected thereby, without water for several days. His experience in India we do not think can settle this question for the African camel; the Indian camel not having the same immense sandy wastes to travel over that the African has, will probably not have accommodated itself to the same deprivations that its African fellow *has to* undergo. Even in horses geographical differences in this respect are noticed. It has been observed as one of the characteristic signs of the true Arab steed at home, that it is contented with less than one quarter of the amount of water required by other breeds. As to the other fact which Mr. Steele thinks has been exaggerated by some, that the camel's hump is a reserve stock which is drawn upon when the animal is deprived of food, we have noticed that an old camel at the Park nearly lost its hump while refusing its food from some digestive trouble, and regained it on resuming feeding.

An interesting observation by Mr. Kettlewell, contained in Mr. Steele's paper, may explain why the camel requires so little water, and, in our opinion, also why pleurisy, a disease usually due to surface chilling, especially when the surface is moist from perspiration, is rare in this animal. He found that the skin of the camel is deficient in sudariporous glands and ducts.

